# Image processing methods to elucidate spatial characteristics of retinal microglia after optic nerve transection

**DOI:** 10.1038/srep21816

**Published:** 2016-02-18

**Authors:** Yudong Zhang, Bo Peng, Shuihua Wang, Yu-Xiang Liang, Jiquan Yang, Kwok-Fai So, Ti-Fei Yuan

**Affiliations:** 1 School of Computer Science and Technology, Nanjing Normal University, Nanjing, China; 2 Institute of Biomedical and Health Engineering, Shenzhen Institutes of Advanced Technology, Chinese Academy of Sciences, Shenzhen, China; 3 State Key Laboratory of Brain and Cognitive Sciences, The University of Hong Kong, Hong Kong, China; 4 Jiangsu Key Laboratory of 3D Printing Equipment and Manufacturing, Nanjing, Jiangsu 210042, China; 5 GHM Institute of CNS Regeneration, Jinan University, Guangzhou, China; 6 Department of Ophthalmology, Li Ka Shing Faculty of Medicine, The University of Hong Kong, China; 7 School of Biomedical Sciences, Li Ka Shing Faculty of Medicine, The University of Hong Kong, Hong Kong, China; 8 Ministry of Education CNS Regeneration International Collaborative Joint Laboratory, Jinan University, Guangzhou, China; 9 School of Psychology, Nanjing Normal University, Nanjing, China

## Abstract

Microglia are the mononuclear phagocytes with various functions in the central nervous system, and the morphologies of microglia imply the different stages and functions. In optical nerve transection model of the retina, the retrograde degeneration of retinal ganglion cells induces microglial activations to a unique morphology termed rod microglia. A few studies described the rod microglia in the cortex and retina; however, the spatial characteristic of rod microglia is not fully understood. In this study, we built a mathematical model to characterize the spatial trait of rod microglia. In addition, we developed a Matlab-based image processing pipeline that consists of log enhancement, image segmentation, mathematical morphology based cell detection, area calculation and angle analysis. This computer program provides researchers a powerful tool to quickly analyze the spatial trait of rod microglia.

Microglia detect injury signals in the central nervous system (CNS) and get activated. In the brain they undergo different stages of activation, which can be classified according to the morphological and immune-reactive diversities^1^. Retinal microglia represent a different population of microglial cells than brain microglia, given the distinct interaction partners and the microenvironments. Investigating retinal microglia activation and de-activation helps to understand the cellular mechanism underlying neurodegenerative diseases in the retina^2^,^3^, and therefore the relevant therapies for neuroprotection.

Optic nerve transection (ONT) is a well-established model to induce progressive retinal ganglion cell (RGC) loss, and triggers retinal microglial activation predominantly at the ganglion cell layer (GCL)/nerve fiber layer (NFL)^4^,^5^. These activated retinal microglial cells undergo proliferation, and express progenitor cell markers such as nestin, Vimentin, and NG2^6^,^7^. In previous studies^4^,^5^,^7^, retinal sections were routinely used for immunohistochemical staining while the global views at the GCL/NFL are usually lacking. Recently, with whole-mount retina immunostaining, we described the appearance of rod microglia cells in ganglion cell layer of retina from 7 days, peaking at 3 weeks and disappearing after 6 weeks of optic nerve transection^8^.

Microglial cells show complicated morphologies and are therefore difficult to quantify. Automatic image processing with computerized software allows faster and high-throughput screening of microglia changes, based on extraction of specific type of microglia cells from immunostaining images. Here, we developed a computerized approach to quantify and to measure the microglia dynamics based on images obtained from whole-mount retina staining.

## Materials and Methods

### Ethics

All animal experiments were conducted according to the Guide of the Committee of Use of Laboratory Animals for Teaching and Research (CULATR) of The University of Hong Kong, Animal Ethics Committee of Shenzhen Institutes of Advanced Technology at Chinese Academy of Sciences, and Nanjing Normal University Animal Research Ethic Committee. The study has been approved by Animal Research Ethic Committee in The University of Hong Kong, Shenzhen Institutes of Advanced Technology at Chinese Academy of Sciences and Nanjing Normal University.

### Animals

40 adult male Sprague–Dawley (SD) rats (220–250 g, aged 8–10 weeks) were used in the experiments. The animals were housed with food and water *ad libitum* under 12-hour light/12-hour dark cycle (7:00 AM–7:00 PM). For the surgery, the animals were anesthetized and maintained with muscular injections of a mixture of ketamine (80 mg/kg) and xylazine (8 mg/kg). For ONT, 0.5% alcaine (Alcon-Couvreur, Puurs, Belgium) was applied to the eyes prior to the surgery, and antiseptic eye drops (Tobres [Tobramycin 0.3%], lcon-Couvreur) were used to prevent infection after the procedures. Finadyne (0.025 mg/mL, Sigma) in drinking water was applied for 7 days to relieve the pain after the surgeries when needed. All animals were sacrificed with overdose of pentobarbital at different time points of interest.

### Optic nerve transection (ONT)

For ONT, after the animal was anesthetized by ketamine (80 mg/kg) and xylazine (8 mg/kg), the posterior pole of the eye was exposed through a superior temporal intra-orbital approach. The eyelid was lifted up using a suture, and bulbar conjunctiva was cut coronally to expose the superior extraocular muscles. By lifting up the muscles using forceps, the intraorbital portion of the optic nerve (ON) was exposed and its dura sheath was opened longitudinally. A complete transection was made to the ON at 1.5 mm posterior to the optic disc as previously described. Care was taken to maintain the blood supply to the retina.

The animals were sacrificed 1, 3, 7, 14, 21 days, 6 weeks and 8 weeks after ONT, and retinas were harvested for whole-mount immunohistochemistry.

### Retinal whole-mount immunohistochemistry

The retinas were fixed in 4% PFA (Sigma) at room temperature for 1 hour, followed by PBS (Sigma) wash. Then, the retinas were blocked by 0.5% triton X-100, 1% bovine serum albumin (BSA, Sigma) and 10% normal goat serum (Jackson) in PBS at room temperature for 2 hours. After that, they were incubated in a diluted primary antibody solution overnight at room temperature. After sequential PBS wash, they were incubated in secondary antibody solution at room temperature for 2 hours. Finally, the retinas were washed in PBS and mounted with fluorescein mounting medium (Dako). The antibodies used included rabbit anti-Iba1 (1:500, Wako), goat anti-rabbit IgG Alexa Fluor 488 (1:200, Life Technology).

### Microscopy

The whole-mount retinas were visualized with a Zeiss Axiophot epi-fluorescence microscope; confocal images (2048 × 2048) were captured by Carl Zeiss LSM 700/710 laser scanning microscopes for analysis. Microglial cells were rendered by green pseudo-color. Both bright field and fluorescent images were collected sequentially.

### Preprocessing of confocal images

All image analysis programs were developed in-house on the platform of Matlab 2015a. The image processing toolbox was used to accelerate the program development.

All confocal images of microglial cells were rendered by fluorescent immunohistochemistry staining. The original image sizes are of 2048 × 2048. Three color channels represent Red, Green, and Blue. The data are stored in uint16 format in the range of [0, 65535].

We first transform the data format from uint16 to double, so as to meet the precision requirement of digital image processing. This is accomplished using the “im2double” command.

The color image was then transformed to a grayscale image because the grayscale version contains enough information. Cells take larger grayscale values, and backgrounds take smaller values. This process is accomplished by the “rgb2gray” command.

Next, the log enhancement was applied to improve visual clarity. The log enhancement was achieved by the “log” command.

After log enhancement, the grayscale values of the image may be compressed into a small-scale range. Therefore, we needed to normalize the intensity values to the range of [0, 1]. This is carried out by the “mat2gray” command. After this step, the intensities of the images are normalized to the range from 0.0 (black) to 1.0 (white).

### Segmentation

Segmentation partitions the confocal image into two parts (cell and background). This can simplify the image representation. Suppose the original image is *I* and the threshold is *T*, then the segmented image *S* is a binary image with pixel values either 0 (background) or 1 (Microglia cell).


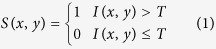


Otsu’s method^9^ is employed to obtain the optimal threshold. It assumes the image contains two class of pixels with bi-modal histogram, then it can obtain the optimal threshold that separates the two classes, in the criterion that the intra-class variance is minimal and inter-class variance is maximal^10^. Again, we used the “graythresh” command to accomplish this process.

### Mathematical morphology based cell detection

Mathematical morphology (MM) is a computer-science related technique to process geometric structures based on set theory, topology, etc. By MM the cell detection problem can be transformed to a connected component (CC) detection problem.

CCs are detected using the command “bwconncomp”. This function finds all the CCs in the segmented image *S*. We manually removed CCs with very small areas that reflect noise. Each CC corresponds to a microglia cell. Note that sometimes several cells overlap with each other. In this case, we resort to manual segmentation. If the overlap is severe, we omit these cells from the following analysis.

Pixel connectivity is the way each pixel relates to its neighbors. In the 4-connected neighbor approach, the neighboring pixels are considered to be those which share an edge with the given pixel. In this system, pixel (x, y) has neighbors (x ± 1, y) and (x, y ± 1). In this work, we employed the 8-connected neighbor approach, where a pixel is considered a neighbor if it touches either the edges or the corners of a given pixel. In this system, pixel (x, y) has neighbors (x ± 1, y), (x, y ± 1), and (x ± 1, y ± 1).

### Ellipse approximation of microglia

After obtaining all the CCs of the microglia image, we generated an optimal ellipse to approximate each CC. The general parametric form of an ellipse in trigonometry is


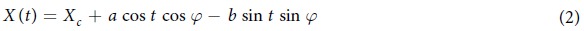



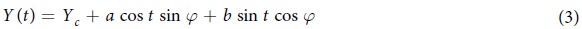


where *a* and *b* represents the semi-major and semi-minor axis, respectively. (*X*_*c*_, *Y*_*c*_) represents the center of the ellipse, *φ* is the angle between the horizontal direction and the major axis of the ellipse. In all, an ellipse has five parameters (*a*, *b*, *X*_*c*_, *Y*_*c*_, *φ*). We vary the values of these parameters so that the ellipse *E* approximates the microglia region CC.


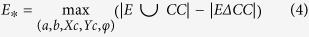


where ∩ is the intersection operator, Δ represents the symmetric difference, and || is the cardinality operator, meaning the number of elements in the set. From the above equation, the criterion is that we expect to maximize the overlapping areas of *E* and CC, while reducing the extra area of *E* apart from CC.

### Area Calculation

The area of CC is calculated as the area of microglia cell. A simple method is to summarize the number of pixels. Considering different patterns of pixels should be weighted differently, we employed an advanced technique that counts the areas of 2 × 2 neighborhood following six rules:

Neighborhood with zero “1” pixel → Area = 0

Neighborhood with one “1” pixel → Area = 1/4

Neighborhood with two adjacent “1” pixels → Area = 1/2

Neighborhood with two diagonal “1” pixels → Area = 3/4

Neighborhood with three “1” pixels → Area = 7/8

Neighborhood will all “1” pixels → Area = 1

### Angle analysis of microglia

Remember the parameter φ, defined as the orientation of the microglial cell. Here it has another physical meaning in that it represents the orientation of the microglial cell within the range of [0, 180] degrees. We obtain the *φ* values of all CCs, and then implemented a count analysis by the “histogram” command.

## Results

### Image Enhancement

**Figure 1:**
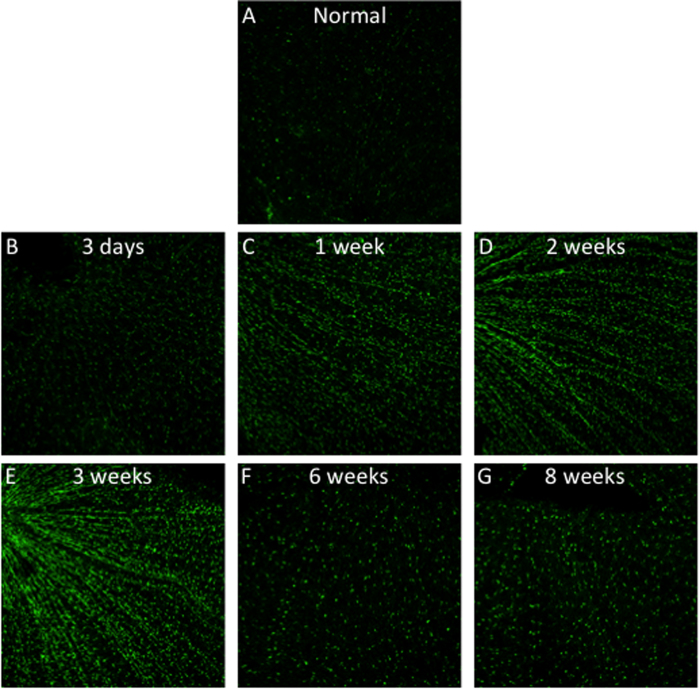
Retinal whole-mount immunostaining of microglial cells at different time points after optic nerve transection (ONT).

Microglia were labeled by microglial specific marker Iba1^11^ and visualized in green. The log enhanced images are shown in Fig. 1.

### Image Segmentation and CC detection

**Figure 2:**
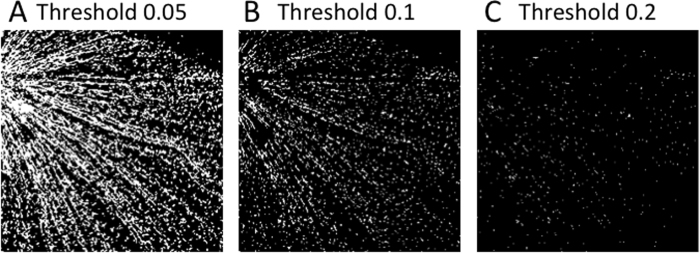
Image Segmentation by the best threshold by grid-search method (**A**) Threshold = 0.05; (**B**) Threshold = 0.1; (**C**) Threshold = 0.2.

0.1 was found by Otsu’s method. To further validate it, we changed the threshold to 0.05 and 0.2. First, we found setting threshold to 0.05 could link separate cells, and make cells aligned into beams (Fig. 2A). On the other hand, setting threshold to 0.2 cannot preserve the shape of individual cells (Fig. 2C). In contrast, assigning 0.1 to the threshold practically made the optimal segmentation (Fig. 2B).

Next, we detected microglial cells from each image by detecting all CCs. Note that the CC detection results still suffer from overlapping problem, which should be fixed by manual check.

### Area Calculation

**Figure 3:**
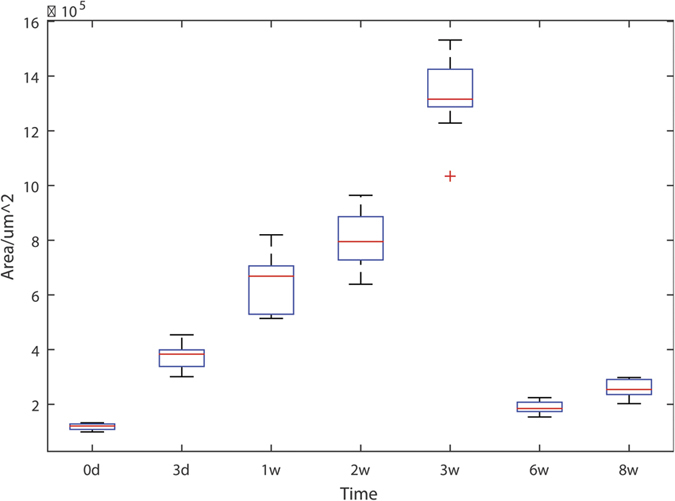
Box plot of microglial cell areas against time.

We calculated areas of rod microglia. To avoid randomness, we select 7 or 8 images at each time step, and average the cell areas. The microglial cell areas significantly increased since 3 days after ONT (Fig. 3). And they continuously increased to three weeks after ONT (Fig. 3). In contrast, the microglial cell areas were declined to normal level at both 6 and 8 weeks after ONT. The result indicates microglia activate from 3 days to 3 weeks after ONT and return to the resting phenotype after 6 weeks.

### Angle Calculation

**Figure 4:**
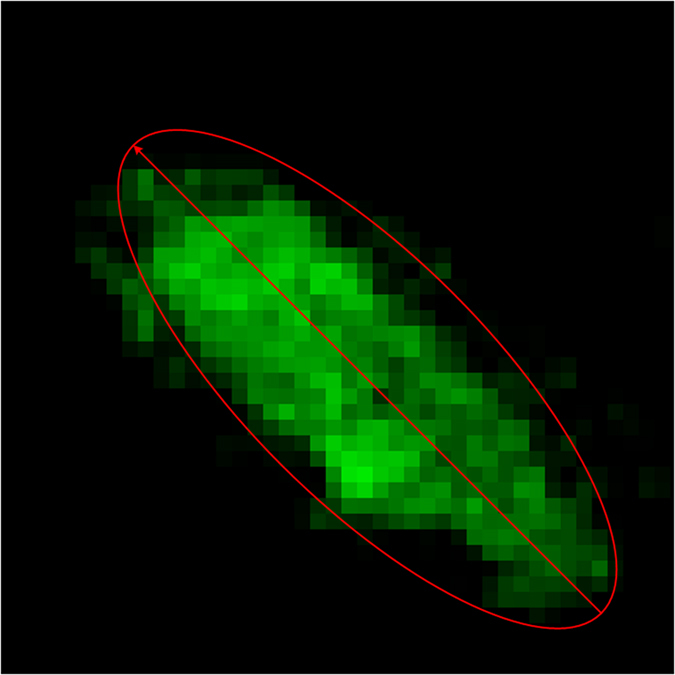
Illustration of how to calculate the angle of a cell.

Rod microglia get aligned to each other, which is one of the major characteristics distinguishing rod microglia from amoeboid microglia^12^. We utilized computer program to analyze the alignment of microglia. The angle of each cell was calculated by the following method. First we generated a minimal ellipse to cover the cell. Then, the orientation of the long axis of this ellipse was regarded as the cell angle. Thus the angle falls within the range of [0 180] in unit of degree. Figure 4 shows an example of how to calculate the angles from a cell.

### Angle Histogram

**Figure 5:**
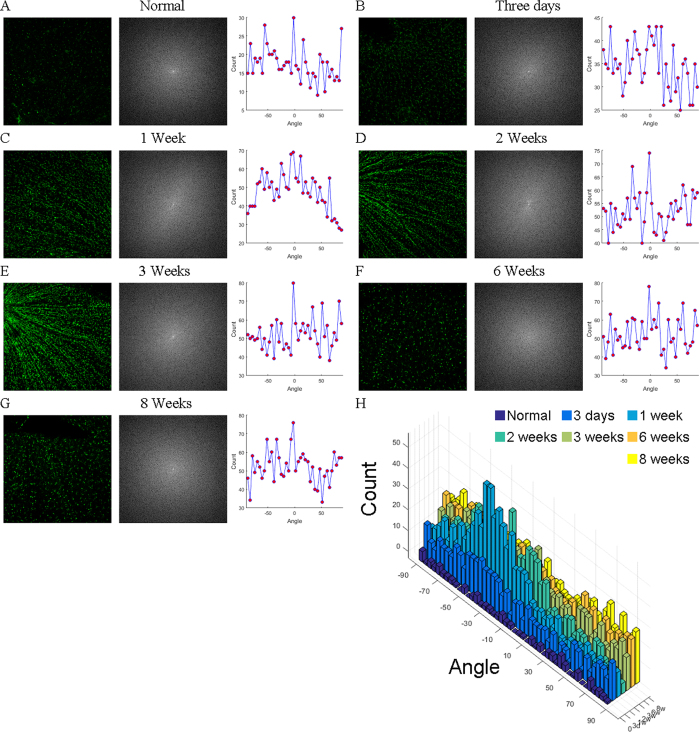
Angle histogram versus time. The angle distributions of microglia in normal retinas (**A**), and retinas of 3 days (**B**), 1 week (**C**), 2 weeks (**D**), 3 weeks (**E**), 6 weeks (**F**) and 8 weeks (**G**) after optic nerve transection. H Compares microglial angles of normal retina and the retinas after optical nerve transection.

Then we counted the number of all microglia in each image (from 0 to 180 with each bin of one degree), and showed the results in Fig. 5. The middle column of each panel represents the frequency space that was obtained by 2D fast Fourier transform with image enhancement by log algorithm. The last column represents the angle histogram. The value of each bin was average among 7 or 8 pictures for each time point we analyzed. In normal retina, the microglial angle was randomly distributed, indicating microglia did not form rod phenotype by the time point (Fig. 5A). 3 days after ONT, the microglia number increased as the microglial occupied areas increased (Fig. 3); however, the microglial angle was still evenly distributed (Fig. 5B), indicating by that time, activated microglia still did not turn into rod phenotype. In contrast, by 1 week after ONT, the microglial angles were accumulated into [−50, 0] degree (Fig. 5C). The angle preference indicates most of microglia got aligned to each other forming certain angles. The angle preference lasted at least to three weeks after ONT (Fig. 5 D,E). Then, the microglial angles turned to evenly distributed after six weeks post surgery (Fig. 5 F,G), indicating the retinal microglia turn back to the resting state by six weeks after ONT, which is consistent with the microglial area calculation (Fig. 3). Figure 5H shows more detailed and intuitive information of the angle distribution.

## Discussion

Glial cells are important components in the central nervous system. Microglia cells participate in neural development, synaptic plasticity and clearance of tissue debris after injury. The environmental cues mediate the functions of microglia; meanwhile, morphologies of microglia are associated with diverse functions they exert^1^,^13^. To a certain extent, the morphology reveals the function of microglia, allowing researchers to estimate the pathological stage of neurodegenerative disorders.

As a newly recognized phenotype, rod microglia show their unique morphology and alignment in some neurodegenerative models^8^,^14^,^15^. However, the definition of morphology is subjective, which makes rod microglial phenotype ambiguous. In our study, we utilized the computer program to quantitatively analyze the morphology of microglia, and found rod microglial cells aligned at a certain orientation. The definitive description by computer processing provides an unambiguous and objective criterion of rod microglia phenotype. In traditional biomedical studies, the analyses largely rely on the experienced researchers, which are time consuming. In contrast, computerized analyses do not rely on the analyzers. The built-in algorithms by the program can accomplish the analytical tasks automatically. Moreover, the computer program may allow high throughput analyses with more precise details.

On the other hand, there are still some limitations in this study. First, we only analyzed the angle of activated microglial cells. The cell angle distribution is a hall mark of rod microglia in the retina; however, the morphology of microglia is also an important factor reflecting their function. In resting state, they show ramified morphology with small cell bodies and long processes. While in response pathogens or insults, they show amoeboid morphology with large cell bodies and stout processes. Using computer programs to analyze the morphological characteristics will be useful to help researchers and clinicians evaluate the neuroinflammation level. Second, our computer program requires manual manipulations to segment overlapping cells. If the overlapping degree is severe, we may need to exclude those cells for analyses.

In summary, by using computer program to analyze microglia, we did not only provide a method in this specific study per se, but also intended to propose a promising idea for biomedical studies in the future.

## Additional Information

**How to cite this article**: Zhang, Y. *et al.* Image processing methods to elucidate spatial characteristics of retinal microglia after optic nerve transection. *Sci. Rep.* **6**, 21816; doi: 10.1038/srep21816 (2016).
